# Supplementary material for: Identification of Freezing-Responsive microRNAs and Their Targets in Chinese Jujube by Small RNA and Degradome Sequencing
Source: Int J Mol Sci. 2026 Sep 4;27(17):7912. doi: 10.3390/ijms27177912 (PMC13566890; doi:10.3390/ijms27177912)
Supplement: Supplementary file 1 [file ijms-27-07912-s001.zip › Supplementary Material S2/novel_miR_200.pdf]

Provisional ID	:	novel_miR_200
Score total	:	1458.9
Score for star read(s)	:	3.9
Score for read counts	:	1452.6
Score for mfe	:	0.8
Score for randfold	:	1.6
Score for cons. seed	:	
Total read count	:	2861
Mature read count	:	2054
Loop read count	:	5
Star read count	:	802

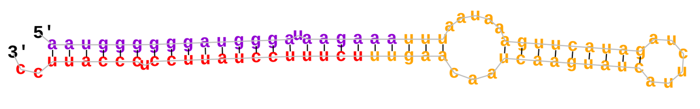

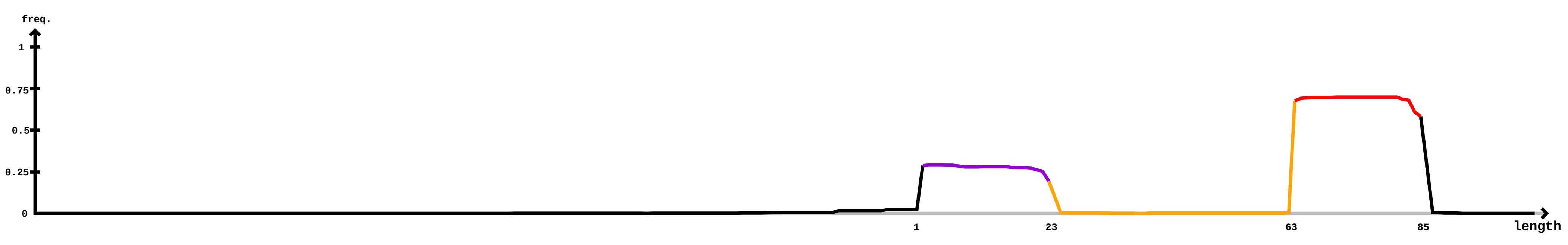

Star Mature

```
5'- gcagacaacuuuuuauauggucggucccaaaaucuuccucuugaaaacccuagcaaagcaaauuugcaaaaacgcauaucggcagagaaaaaaaaauugaaaacagagagacgggaaagguagcaaagaguuaggagguuauuaggaaugggggaugggauaagaaauuuaauaaaguucauagaucuuacuaugaacuaacaaguuucuuuccuauuccucccauuccauucaauuuccuaauuuccu -3' obs
    gcagacaacuuuuuauauggucggucccaaaaucuuccucuugaaaacccuagcaaagcaaauuugcaaaaacgcauaucggcagagaaaaaaaaauugaaaacagagagacgggaaagguagcaaagaguuaggagguuauuaggaaugggggaugggauaagaaauuuaauaaaguucauagaucuuacuaugaacuaacaaguuucuuuccuauuccucccauuccauucaauuuccuaauuuccu exp
    ...(((.(((........)))..)))((....((.((((((..........(((((.....))))).....((......))...................)))).)).))....)).......((((((((((((....((((((((((((((((.(((((((((.....(((((((((......)))))))))...))))))))))))))))).)))))))).....)))))))))))))...
```

read	reads	mm	sample
ucggcagagaaaaaaaaauuga	2	0	LCK-3
aacagagagacgggaaagg	3	0	LCK-3
aagguagcaaagaguuaggagguuauuagg	1	0	JT-3
aagguagcaaagaguuaggagguuauuagg	2	0	JT-2
uagcaaagaguuaggagguua	1	0	LT-1
uagcaaagaguuaggagguuauuagg	2	0	LT-2
uagcaaagaguuaggagguuauuagg	2	0	JCK-2
uagcaaagaguuaggagguuauuagg	1	0	JCK-3
agcaaagaguuaggagguuauuagg	1	0	JCK-2
agcaaagaguuaggagguuauuagg	1	0	LT-2
agcaaagaguuaggagguuauuagg	2	0	LT-3
caaagaguuaggagguuauuaggaaug	1	0	LT-3
uaggagguuauuaggaaug	1	0	JCK-1
uaggagguuauuaggaauggggg	1	0	JCK-3
aggagguuauuaggaauggg	8	0	JT-2
aggagguuauuaggaauggg	10	0	JT-3
aggagguuauuaggaaugggC	1	1	JCK-2
aggagguuauuaggaaugggg	8	0	LCK-3
aggagguuauuaggaaugggg	4	0	JCK-2
aggagguuauuaggaaugggg	1	0	LT-2
auuaggaaugggCggaugggа	1	1	JCK-1
auuaggaaugggCggaugggа	16	1	JT-1
auuaggaaugggggaugggau	1	0	LT-1
auuaggaaugggCggaugggauaagaa	1	1	JT-3
gaaugggggaugggauaaga	1	0	LT-3
aaugggCggaugggauaa	7	1	JT-1
aaugggCggaugggauaa	2	1	JCK-1
aaugggCggaugggauaag	3	1	JT-3
aaugggggaugggauaag	6	0	JT-2
aaugggCggaugggauaag	5	1	JT-2
aaugggggaugggauaag	3	0	LT-2
aaugggCggaugggauaag	1	1	JCK-3
aaugggggaugggauaag	1	0	JCK-2

Star Mature

gcagacaacuuuuuauauggucggucccaaaaucuuccucuugaaaacccuagcaaagcaaauuugcaaaaacgcauaucggcagagaaaaaaaaauugaaaacagagagacgggaaagguagcaaagaguuaggagguuauuaggaauggggggaugggauaagaaauuuaauaaaguucauagaucuuacuaugaacuaacaaguuucuuuccuauuccucccauuccauucaauuuccuaauuuccu

aauggggggaugggauaag	7	0	JT-3
aaugggCggaugggauaaga	8	1	JCK-2
aaugggCggaugggauaaga	10	1	JCK-3
aauggggggaugggauaaga	8	0	JT-1
aauggggggaugggauaaga	1	0	JCK-1
aauggggggaugggauaaga	1	0	LT-1
aauggggggaugggauaaga	3	0	LT-3
aauggggggaugggauaaga	3	0	JCK-2
aauggggggaugggauaagaa	13	0	LT-2
aaugggCggaugggauaagaa	13	1	JT-2
aauggggggaugggauaagaa	8	0	JT-3
aaugggCggaugggauaagaa	25	1	JCK-3
aaugggCggaugggauaagaa	36	1	JCK-2
aauggggggaugggauaagaa	4	0	JT-2
aauggggggaugggauaagaa	14	0	JCK-2
aauggggggaugggauaagaa	3	0	JCK-1
aauggggggaugggauaagaa	7	0	JCK-3
aauggggggaugggauaagaa	5	0	LT-3
aaugggCggaugggauaagaa	16	1	JT-3
aaugggCggaugggauaagaa	16	1	JCK-1
aauggggggaugggauaagaa	2	0	LT-1
aauggggggaugggauaagaa	6	0	LCK-3
aauggggggaugggauaagaaU	1	1	JCK-1
aauggggggaugggauaagaaa	10	0	JT-1
aauggggggaugggauaagaaa	9	0	LT-3
aauggggggaugggauaagaaU	1	1	JCK-3
aauggggggaugggauaagaaa	14	0	JCK-3
aauggggggaugggauaagaaa	12	0	LT-1
aaugggCggaugggauaagaaa	73	1	JT-1
aauggggggaugggauaagaaa	5	0	JT-2
aauggggggaugggauaaAaaa	1	1	JCK-2
aaugggCggaugggauaagaaa	66	1	JCK-1
aauggggggaugggauaagaaa	12	0	JCK-1
aaugggCggaugggauaagaaa	62	1	JT-3
aauggggggaugggauaagaaC	2	1	LT-3
aauggggggaugggauaagaaa	27	0	JCK-2
aauggggggaugggauaagaaa	14	0	LT-2
aauggggggaugggauaagaaU	2	1	JCK-2
aauggggggaugggauaagaaU	3	1	LCK-3
aauggggggaugggauaagaaa	6	0	JT-3
aaugggCggaugggauaagaaa	35	1	JT-2
aaugggCggaugggauaagaaa	107	1	JCK-2
aaugggCggaugggauaagaaa	83	1	JCK-3
aaugggCggaugggauaagaaau	1	1	JCK-3
aauggggggaugggauaagaaUu	1	1	LT-1
aaugggCggaugggauaagaaau	3	1	JCK-1
aaugggCggaugggauaagaaau	3	1	JCK-2
aaugggCggaugggauaagaaauu	2	1	JCK-3
aaugggCggaugggauaagaaauu	1	1	JCK-1
auggggggaugggauaagaaa	2	0	JCK-1
augggCggaugggauaagaaa	5	1	JCK-2
auggggggaugggauaagaaa	1	0	JCK-3
Cggaugggauaagaaauuuaauaa	1	1	JCK-2
Cggaugggauaagaaauuuaauaa	2	1	JCK-3
ugggauaagaaauuuaauaaag	1	0	JCK-3
ugggauaagaaCuuuaauaaag	1	1	JCK-1
ugggauaagaaauuuaauaaaguuca	2	0	LCK-3
gaucuuacuaugaacuaacaaguA	5	1	LCK-3
uucuuuccuauuccucccauuc	5	0	LT-2
uucuuuccuauuccucccauuc	1	0	JCK-3
Cucuuuccuauuccucccauucc	1	1	JCK-3
Cucuuuccuauuccucccauucc	1	1	JCK-2
ucuuuccuauuccuccca	5	0	LT-1
ucuuuccuauuccuccca	15	0	LT-3
ucuuuccuauuccucccU	2	1	LT-3
ucuuuccuauuccuccca	1	0	JCK-2
ucuuuccuauuccuccca	7	0	JT-3
ucuuuccuauuccuccca	1	0	JCK-3
ucuuuccuauuccuccca	3	0	JT-2
ucuuuccuauuccuccca	3	0	LCK-3

```
                                                                                                                                                                                 Star                                                           Mature
gcagacaacuuuuuauauggucggucccaaaaucuuccucuugaaaacccuagcaaagcaaauuugcaaaaacgcauaucggcagagaaaaaaaaauugaaaacagagagacgggaaagguagcaaagaguuaggagguuauuaggaauggggggauggggauaagaaauuuaauaaaguucauagaucuuacuaugaacuaacaaguuucuuuccuauuccucccauuccauucaauuuccuaauuuccu
.................................................................................................................................................................................................................ucuuuccuauuccucccau.......................   7     0   LCK-3
.................................................................................................................................................................................................................ucuuuccuauuccucccau.......................   2     0   LT-1
.................................................................................................................................................................................................................ucuuuccuauuccucccUu.......................   1     1   LT-1
.................................................................................................................................................................................................................ucuuuccuauuccucccau.......................   3     0   LT-2
.................................................................................................................................................................................................................ucuuuccuauuccucccau.......................   4     0   LT-3
.................................................................................................................................................................................................................ucuuuccuauuccucccauu......................   29    0   JCK-2
.................................................................................................................................................................................................................ucuuuccuauuccucccauu......................   16    0   JCK-1
.................................................................................................................................................................................................................ucuuuccuauuccucccauu......................   41    0   LCK-3
.................................................................................................................................................................................................................ucuuuccuauuccucccauu......................   25    0   JCK-3
.................................................................................................................................................................................................................ucuuuccuauuccucccCuu......................   1     1   LT-2
.................................................................................................................................................................................................................ucuuuccuauuccucccauu......................   31    0   LT-2
.................................................................................................................................................................................................................ucNuuccuauuccucccauu......................   1     1   LT-2
.................................................................................................................................................................................................................ucuuuccuauuccucccauu......................   27    0   LT-3
.................................................................................................................................................................................................................ucuGuccuauuccucccauu......................   1     1   JCK-1
.................................................................................................................................................................................................................ucuuuccuCuuccucccauu......................   1     1   LT-3
.................................................................................................................................................................................................................ucuNuccuauuccucccauu......................   1     1   JCK-1
.................................................................................................................................................................................................................ucuuuccuauuccucccauG......................   1     1   JCK-1
.................................................................................................................................................................................................................ucuuuccuauuccucccauu......................   20    0   LT-1
.................................................................................................................................................................................................................ucuuuccuauuccucccauuc.....................   1     0   JT-3
.................................................................................................................................................................................................................ucuuuccuauuccucccauuc.....................   8     0   LT-3
.................................................................................................................................................................................................................ucuuuccuauuccucccauuc.....................   12    0   JCK-1
.................................................................................................................................................................................................................ucuuuccuauuccucccauuc.....................   6     0   LT-1
.................................................................................................................................................................................................................ucuuuccuauuccucccauuc.....................   12    0   LT-2
.................................................................................................................................................................................................................ucuuuccuauuccucccauuc.....................   1     0   JT-2
.................................................................................................................................................................................................................ucuuuccuauuccucccauuc.....................   14    0   JCK-2
.................................................................................................................................................................................................................ucuuuccuauuccucccCuuc.....................   1     1   JCK-2
.................................................................................................................................................................................................................ucuuuccuauuccucccUuuc.....................   1     1   LT-1
.................................................................................................................................................................................................................ucuuuccuauuccucccauuA.....................   1     1   JCK-1
.................................................................................................................................................................................................................ucuuuccuauuccucccauuc.....................   11    0   JCK-3
.................................................................................................................................................................................................................uNuuuccuauuccucccauucc....................   1     1   JCK-2
.................................................................................................................................................................................................................ucuuuccuauuccucccCuucc....................   1     1   JCK-1
.................................................................................................................................................................................................................ucuCuccuauuccucccauucc....................   1     1   JCK-2
.................................................................................................................................................................................................................ucuuuccuauuccucccauucc....................   64    0   LCK-3
.................................................................................................................................................................................................................uUuuuccuauuccucccauucc....................   2     1   JCK-1
.................................................................................................................................................................................................................ucuuuccuauucAucccauucc....................   1     1   JCK-3
.................................................................................................................................................................................................................ucuuucUuauuccucccauucc....................   1     1   JCK-2
.................................................................................................................................................................................................................ucuuuccuCuuccucccauucc....................   1     1   JCK-1
.................................................................................................................................................................................................................ucuuuccuauuccucccCuucc....................   1     1   LCK-3
.................................................................................................................................................................................................................ucuuuccuauuccucccauuUc....................   1     1   JCK-1
.................................................................................................................................................................................................................ucuuuccuauuccucccauucc....................   13    0   JT-3
.................................................................................................................................................................................................................ucuuuccuauuccucccauucc....................   22    0   JT-1
.................................................................................................................................................................................................................ucuuuccuauuccucccauucU....................   1     1   JCK-3
.................................................................................................................................................................................................................ucuuuccuCuuccucccauucc....................   1     1   LT-1
.................................................................................................................................................................................................................ucuuuccCauuccucccauucc....................   1     1   JCK-2
.................................................................................................................................................................................................................ucuuuccuauucUucccauucc....................   2     1   JCK-2
.................................................................................................................................................................................................................ucuuuccCauuccucccauucc....................   1     1   JT-1
.................................................................................................................................................................................................................ucuuuccuauuccucccCuucc....................   1     1   LT-2
.................................................................................................................................................................................................................uUuuuccuauuccucccauucc....................   1     1   JCK-2
.................................................................................................................................................................................................................ucuuuccuauuUcucccauucc....................   1     1   JCK-3
.................................................................................................................................................................................................................ucuuuccuauuccucccauuNc....................   2     1   JCK-1
.................................................................................................................................................................................................................ucuuuccuauuccucccauucc....................   388   0   JCK-3
.................................................................................................................................................................................................................ucuuuccuCuuccucccauucc....................   3     1   JCK-3
.................................................................................................................................................................................................................ucuuuccuauuccucccauucc....................   140   0   LT-3
.................................................................................................................................................................................................................ucuuuccuauucAucccauucc....................   1     1   LT-3
.................................................................................................................................................................................................................ucuuuccuauuccucccCuucc....................   2     1   JCK-2
.................................................................................................................................................................................................................ucuuuccuauuccucccauucc....................   123   0   LT-1
.................................................................................................................................................................................................................ucuuuccuauuccucccauucc....................   87    0   LT-2
.................................................................................................................................................................................................................uUuuuccuauuccucccauucc....................   2     1   JCK-3
.................................................................................................................................................................................................................ucuuuccuauuccucccauucc....................   3     0   JT-2
.................................................................................................................................................................................................................ucuuuccuauuccucccauucc....................   527   0   JCK-2
.................................................................................................................................................................................................................ucuuuccuauuAcucccauucc....................   1     1   LT-3
.................................................................................................................................................................................................................ucuuuccuauuccucccauucc....................   250   0   JCK-1
.................................................................................................................................................................................................................ucuuuccuauuccucccCuucca...................   1     1   JT-1
.................................................................................................................................................................................................................ucuuuccuauuccucccauucca...................   2     0   LT-3
.................................................................................................................................................................................................................ucuuuccuauuccucccauucca...................   11    0   JT-1
.................................................................................................................................................................................................................ucuuuccuauuccucccauucca...................   2     0   LT-1
.................................................................................................................................................................................................................ucuuuccuauuccucccauucUa...................   1     1   LT-3
.................................................................................................................................................................................................................ucuuuccuauuccucccauuccC...................   4     1   LT-2
.................................................................................................................................................................................................................ucuuuccuauuccucccauuccau..................   1     0   JCK-1
..................................................................................................................................................................................................................cuuuccuauuccucccauu......................   1     0   JCK-3
```

```
                                                                                                                                                                                       Star                                                                  Mature
gcagacaacuuuuuauauggucggucccaaaaucuuccucuugaaaacccuagcaaagcaaauuugcaaaaacgcauaucggcagagaaaaaaaaauugaaaacagagagacgggaaagguagcaaagaguuaggagguuauuaggaauggggggaugggauaagaaauuuaauaaaguucauagaucuuacuaugaacuaacaaguuucuuuccuauuccucccauuccauucaauuuccuaauuuccu
.................................................................................................................................................................................................................cuuuccuauuccucccauu......................     11    0    LCK-3
.................................................................................................................................................................................................................cuuuccuauuccucccauuc.....................     1     0    LT-3
.................................................................................................................................................................................................................cuuuccuauuccucccauuU.....................     3     1    LT-2
.................................................................................................................................................................................................................cuuuccuauuccucccauucc....................     2     0    LT-1
.................................................................................................................................................................................................................cuuuccuauuccucccauucc....................     3     0    JCK-3
.................................................................................................................................................................................................................cuuuccuauuccucccauucc....................     4     0    JCK-1
.................................................................................................................................................................................................................cuuuccuauuccucccauucc....................     5     0    LT-3
.................................................................................................................................................................................................................cuuuccuauuccucccauucc....................     10    0    JCK-2
.................................................................................................................................................................................................................cuuuccuauuccucccauuccauu.................     4     0    LT-2
..................................................................................................................................................................................................................uuuccuauuccucccauu......................     2     0    JCK-3
..................................................................................................................................................................................................................uuuccuauuccucccauu......................     1     0    JCK-1
..................................................................................................................................................................................................................uuuccuCuuccucccauucc....................     1     1    LT-3
..................................................................................................................................................................................................................uuuccuauuccucccauucc....................     1     0    LT-3
..................................................................................................................................................................................................................uuuccuauuccucccauucc....................     1     0    LT-1
..................................................................................................................................................................................................................uuuccuauuccucccauucc....................     1     0    JCK-3
..................................................................................................................................................................................................................uuuccuauuccucccauucc....................     3     0    JCK-1
...................................................................................................................................................................................................................uuccuauuccucccauucc....................     1     0    LT-3
...................................................................................................................................................................................................................uuccuauuccucccauuccaC..................     2     1    JCK-2
...................................................................................................................................................................................................................uuccuauuccucccauuccauu.................     1     0    JCK-3
...................................................................................................................................................................................................................uuccuauuccucccauuccauu.................     2     0    JCK-1
.......................................................................................................................................................................................................................uauuccucccauuccauucaa..............     1     0    JCK-3
.......................................................................................................................................................................................................................uauuccucccauuccauucaa..............     2     0    JCK-2
.......................................................................................................................................................................................................................uauuccucccauuccauucaa..............     2     0    JCK-1
```
